# Supplementary material for: Recombinant GPEHT Fusion Protein Derived from HTLV-1 Proteins with Alum Adjuvant Induces a High Immune Response in Mice
Source: Vaccines (Basel). 2023 Jan 3;11(1):115. doi: 10.3390/vaccines11010115 (PMC9865465; doi:10.3390/vaccines11010115)
Supplement: Supplementary file 1 [file vaccines-11-00115-s001.zip › vaccines-2019786-supplementary.pdf]

Supplementary S1. The densitometry intensity ratio of each band for the SDS-PAGE and Western blot.

A B

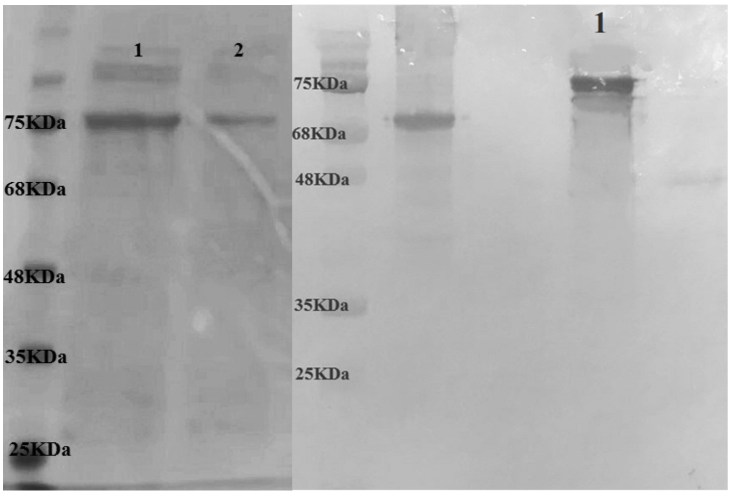

Figure S1 (A)	Intensity	Ratio
blot 1	114288.068	1
blot 2	4321.468	26.44

Figure S1(B)	Intensity	Ratio
blot 1	51276.726	1
